# Survival after lymph node dissection, radiotherapy, and combined treatment in sentinel node–positive stage IIIA Merkel cell carcinoma: A systematic review and meta-analysis

**DOI:** 10.1016/j.jdin.2026.06.025

**Published:** 2026-07-30

**Authors:** Sophia Wasserer, Yannick Foerster, Sarah Preis, Felix Lauffer, Tilo Biedermann, Oana-Diana Persa, Kristine Elisabeth Mayer

**Affiliations:** a Department of Dermatology and Allergy, School of Medicine, Technical University of Munich, Bavarian Cancer Research Center (BZKF), Munich, Germany; b Department of Dermatology and Allergy LMU University Hospital, LMU Medizin, LMU Munich, Bavarian Cancer Research Center (BZKF), Munich, Germany; c Institute for Medical Information Processing, Biometry, and Epidemiology, Pettenkofer School of Public Health LMU Munich, Munich, Germany

**Keywords:** epidemiology, lymphadenectomy, Merkel cell carcinoma, micrometastasis, radiation

## Abstract

**Background:**

Merkel cell carcinoma (MCC) has a high risk of regional lymphatic metastasis. In cases of lymphatic micrometastasis, an evidence-based clinical approach is missing.

**Objective:**

To assess survival outcomes following complete lymph node dissection (CLND), radiation (RTX), or CLND plus RTX (CLND + RTX) in patients with stage IIIA MCC.

**Methods:**

A systematic search was conducted in MEDLINE, Embase, and the Cochrane Library. Pooled survival rates were calculated using random-effects models. Comparative effectiveness of interventions was assessed via network meta-analysis.

**Results:**

Eight studies, including 1825 patients, were analyzed. Pooled 1- and 3-year survival rates (overall survival and MCC-specific survival) following CLND (regardless of RTX) were 93% (95% CI, 89%-96%) and 71% (95% CI, 64%-77%), showing no significant difference compared with patients without CLND. Furthermore, relative risk ratios comparing RTX (regardless of CLND status) with no RTX demonstrated no significant differences for overall survival at either 1 or 3 years. Finally, a network meta-analysis also did not show superiority among CLND only, RTX only, and CLND + RTX in patients with stage IIIA MCC.

**Limitations:**

The limited number of observational studies, data heterogeneity, missing data regarding immunotherapies, and low evidence levels according to the GRADE restrict definitive conclusions.

**Conclusions:**

Findings underscore the need for large, prospective clinical trials to define optimal treatment strategies for patients with stage IIIA MCC.

**PROSPERO number:**

CRD42025636348.

**Capsule Summary**

• Complete lymph node dissection, radiation of the nodal basin (RTX), and the combination of both are approved therapeutic options for patients with stage IIIA Merkel cell carcinoma.
• The network meta-analysis showed comparable survival outcomes with complete lymph node dissection only, RTX only, and complete lymph node dissection plus RTX, suggesting individualized approaches may be reasonable.

## Introduction

Merkel cell carcinoma (MCC) is a rare, aggressive neuroendocrine skin tumor with an increasing global incidence.^1^ Its propensity for early metastasis formation^2^, ^3^, ^4^ contributes to a high mortality rate, exceeding that of melanoma.^5^ Lymphatic drainage is the primary route of metastasis, with occult or clinical lymph node (LN) metastases present in approximately one third of patients at diagnosis.^4^^,^^6^^,^^7^ Consequently, assessment of locoregional spread is recommended for all cases of MCC, regardless of the tumor diameter or thickness.^8^^,^^9^ This evaluation typically involves LN ultrasound or imaging, followed by sentinel lymph node biopsy (SLNB) in clinically inapparent cases.^8^, ^9^, ^10^ SLNB positivity rates are high, exceeding 20% across all age groups.^11^^,^^12^

Despite treatments such as completion lymph node dissection (CLND), radiation (RTX) of the involved nodal basin, or combined modalities,^13^^,^^14^ recurrence rates remain substantial.^7^ In stage IIIA patients, 1-year recurrence rate is 30%.^15^ Five-year survival rates decline from 76% to 42% in patients with LN micrometastasis and to 26% in patients with LN macrometastasis.^4^ Given that CLND has not demonstrated survival benefits in patients with melanoma having lymphatic micrometastasis,^16^, ^17^, ^18^ its prognostic value in patients with MCC is uncertain.

Beyond prognostic implications, both CLND and RTX carry risks of complications, necessitating careful consideration of their indications. CLND exhibits an overall complication rate of approximately 39.3%, which includes severe and potentially long-lasting side effects such as lymphatic drainage disorders, sensory loss, and bacterial infection.^18^, ^19^, ^20^ While RTX is associated with a lower risk of lymphedema,^21^^,^^22^ it can cause fatigue, radiation dermatitis, delayed wound healing, radiation-induced fibrosis, chronic ulceration, and site-specific adverse events. However, evidence guiding the therapeutic management of patients with MCC and a positive SLNB, specifically stage IIIA patients, remains limited. Hence, identifying the best individual treatment strategy presents a clinical challenge.

A large multicenter analysis (*n =* 1049) showed no improvement of progression-free survival (PFS) or disease-specific survival following CLND but reported significant survival benefits with early adjuvant RTX.^6^ Although some studies suggest survival benefits with RTX alone or with combined CLND + RTX compared with CLND alone,^6^^,^^13^ others observed no differences between the 3 interventions.^23^^,^^24^ To synthesize the available data and guide clinical decision making, we conducted a systematic review and meta-analysis evaluating the survival effects of CLND, RTX, and CLND + RTX in patients with MCC and LN micrometastasis.

## Methods

### Search strategy and study selection

This systematic review and meta-analysis was performed according to The Preferred Reporting Items for Systematic Reviews and Meta-Analyses statement^25^ after registration on PROSPERO (CRD42025636348). MEDLINE/PubMed, Embase (via Ovid), and the Cochrane Central Register of Controlled Trials (via Ovid) were searched applying the following terms: (Merkel cell carcinoma OR MCC OR Merkel cell) AND (nodal metastasis OR sentinel OR lymph node metastasis OR stage III OR lymphatic metastasis) AND (surgery OR lymph node dissection OR lymphadenectomy OR radiation OR radiotherapy OR avelumab OR nivolumab OR pembrolizumab OR immune checkpoint). The initial search was run on December 20, 2024, and an update was performed on September 2, 2025. After duplicate removal, SW and KEM independently reviewed titles, abstracts, and full-text manuscripts for study eligibility and solved discrepancies by discussion. Publications in languages other than English and German, case reports, reviews, comments, editorials, guidelines, conference abstracts, cell culture and animal studies, and studies covering the treatment of LN macrometastasis were excluded. For risk-of-bias assessment, SW and KEM independently evaluated the studies using a modified Newcastle-Ottawa Scale Score.^26^^,^^27^ Discrepancies were solved by discussion. If a study met at least 4 of the criteria, sufficient study quality was assumed.^27^

### Data collection, synthesis, and analysis

Data were collected for patients receiving CLND versus no CLND (irrespective of additional RTX), RTX versus no RTX (irrespective of additional CLND), CLND only, RTX only, and CLND + RTX. The following survival rates were extracted where available: 1-, 2-, 3-, and 5-year nodal recurrence-free survival (NRFS), PFS, distant metastasis-free survival, MCC-specific survival (MSS), and overall survival (OS). If the rates were not explicitly stated, WebPlotDigitizer v4.8 was used for data extraction from published Kaplan-Meier curves. To summarize a larger number of studies, combined survival rates from studies reporting on MSS or OS were calculated and compared with OS rates.

For statistical analyses, the meta,^28^ metafor,^29^ metabin,^30^ and netmeta^31^ packages in RStudio v2024.04.2+764.1 were used. First, weighted survival rates for CLND versus no CLND or RTX versus no RTX were calculated as described in a study by Mayer et al.^32^ Differences between subgroups were assessed by χ^2^ tests. *P* values of <.05 were considered statistically significant. Next, the metabin function was used to calculate risk ratios (RRs) of survival for CLND versus no CLND or RTX versus no RTX and pooled estimates using a random-effects (RE) model with inverse-variance weighting. The certainty of evidence was assessed according to the GRADE framework^33^ and added to the summary of findings tables generated using GRADEpro.^34^

Next, we performed a network meta-analysis (NMA) to compare RTX alone and CLND + RTX with CLND alone. Transitivity was assessed based on study population characteristics, such as age and sex (male/female) of the patients, size or stage of primary tumor, site of LN basin, number of positive sentinel lymph nodes, and percentage of immunosuppressed patients. Again, survival (including OS or MSS) rates were summarized as a binary outcome (survived vs not survived). After pairwise comparisons, RRs were calculated. A frequentist NMA using the netmeta package in R was conducted, applying a RE model with restricted maximum likelihood to account for study heterogeneity. The treatment network was displayed using network graphs. For consistency analysis, design-by-treatment interaction models and Q statistics were applied.^35^

For all analyses, pooled RRs with 95% CIs were reported and visualized by forest plots. τ and I^2^ were calculated to assess between-study heterogeneity^36^ and leave-one-out analyses were performed to assess sensitivity.

### Risk of bias and certainty of evidence assessment

Publication bias was assessed using funnel plots and Egger regression tests.^29^ Following Cochrane recommendations for observational studies, KM and SW independently assessed the risk of bias for each outcome using the ROBINS-I tool.^37^ Each study was evaluated across 7 predefined bias domains, with an overall risk-of-bias judgment assigned for each domain ranging from low to serious risk. Additionally, the certainty of evidence for each outcome was evaluated using the GRADE framework,^33^ considering the risk of bias, inconsistency, indirectness, imprecision, and publication bias.

## Results

### Search results and study characteristics

Of 1993 database records, 515 duplicates were removed. After screening of titles and abstracts, 21 full-text articles were assessed for eligibility. Fourteen articles were excluded because they were off-topic or did not contain the relevant data. After including an additional suitable study that was published after the initial screening, 8 observational studies were included in the meta-analysis (Fig 1).^6^^,^^11^^,^^13^^,^^14^^,^^23^^,^^24^^,^^38^^,^^39^ Studies involving patients treated with immune-checkpoint inhibitors (ICIs) were scarce. Among the 37 studies that enrolled stage IIIB patients, only 6 reported ICI treatment (Fig 1).

**Fig 1:**
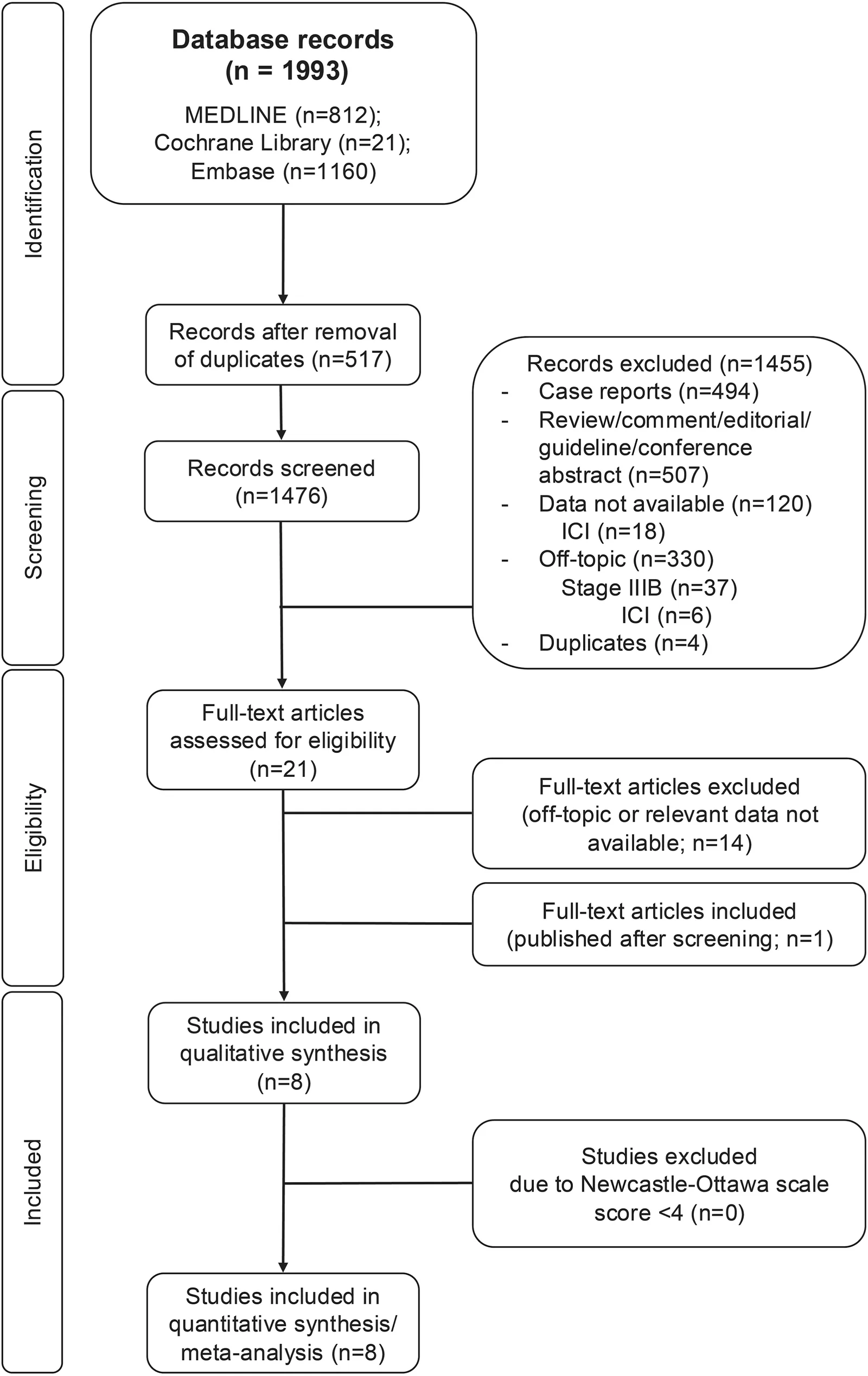
Preferred Reporting Items for Systematic Reviews and Meta-Analyses flow diagram for study identification and selection.

All eight studies included in the meta-analysis were of high methodologic quality, with a modified Newcastle-Ottawa Scale Score of at least 4. In total, the studies reported on 1825 patients with stage IIIA MCC. Eight studies included patients receiving CLND, seven included patients receiving RTX, and six included patients undergoing CLND + RTX.

### Survival after CLND versus no CLND

First, the effects of the interventions on surrogate parameters of local and distant disease control were evaluated. Supplementary Table I (available via Mendeley at https://data.mendeley.com/datasets/27ct2dm8x4/1) summarizes PFS, NRFS, and distant metastasis-free survival for each intervention arm across all studies. RR of NRFS did not differ significantly between CLND versus no CLND at 1 and 3 years (Supplementary Table I). The effects of the intervention CLND were further evaluated regarding survival. OS rates were used when available and analyzed separately or in combination with the MSS rates reported by Lodde et al^6^ and Lee et al.^39^ The weighted 1-year OS and MSS survival rate following CLND (with or without RTX) was 93% (95% CI, 89%-96%) compared with 92% (95% CI, 89%-94%) without CLND, and for OS alone, the survival rate was 94% (95% CI, 88%-98%) compared with 91% (95% CI, 88%-94%), exhibiting no significant difference for OS between the 2 groups (χ^2^ = 2.4, *P* = .1) (Supplementary Fig 1, *A*, available via Mendeley at https://data.mendeley.com/datasets/27ct2dm8x4/1). Study heterogeneity was limited (I^2^ = 53% and I^2^ = 0%, respectively). Weighted 3-year OS and MSS survival rates were 71% (95% CI, 64%-77%) with CLND versus 67% (95% CI, 62%-70%) without CLND, and for OS alone, the survival rate was 67% (95% CI, 59%-75%), compared with 65% (95% CI, 60%-69%), again showing no significant difference for OS between the 2 groups (χ^2^ = 0.07, *P* = .8) (Supplementary Fig 1, *B*, available via Mendeley at https://data.mendeley.com/datasets/27ct2dm8x4/1). However, a 3-year analysis exhibited a larger study heterogeneity (I^2^ = 72% and I^2^ = 0%, respectively). Notably, two studies in each analysis reported MSS. The RR of CLND versus no CLND was compared using a RE model. Hereby, the RR ratio of CLND versus no CLND showed no significant difference for 1- and 3-year pooled OS and MSS survival as well as for OS alone(Figs 2, *A*, *B*, and Table I).

**Fig 2:**
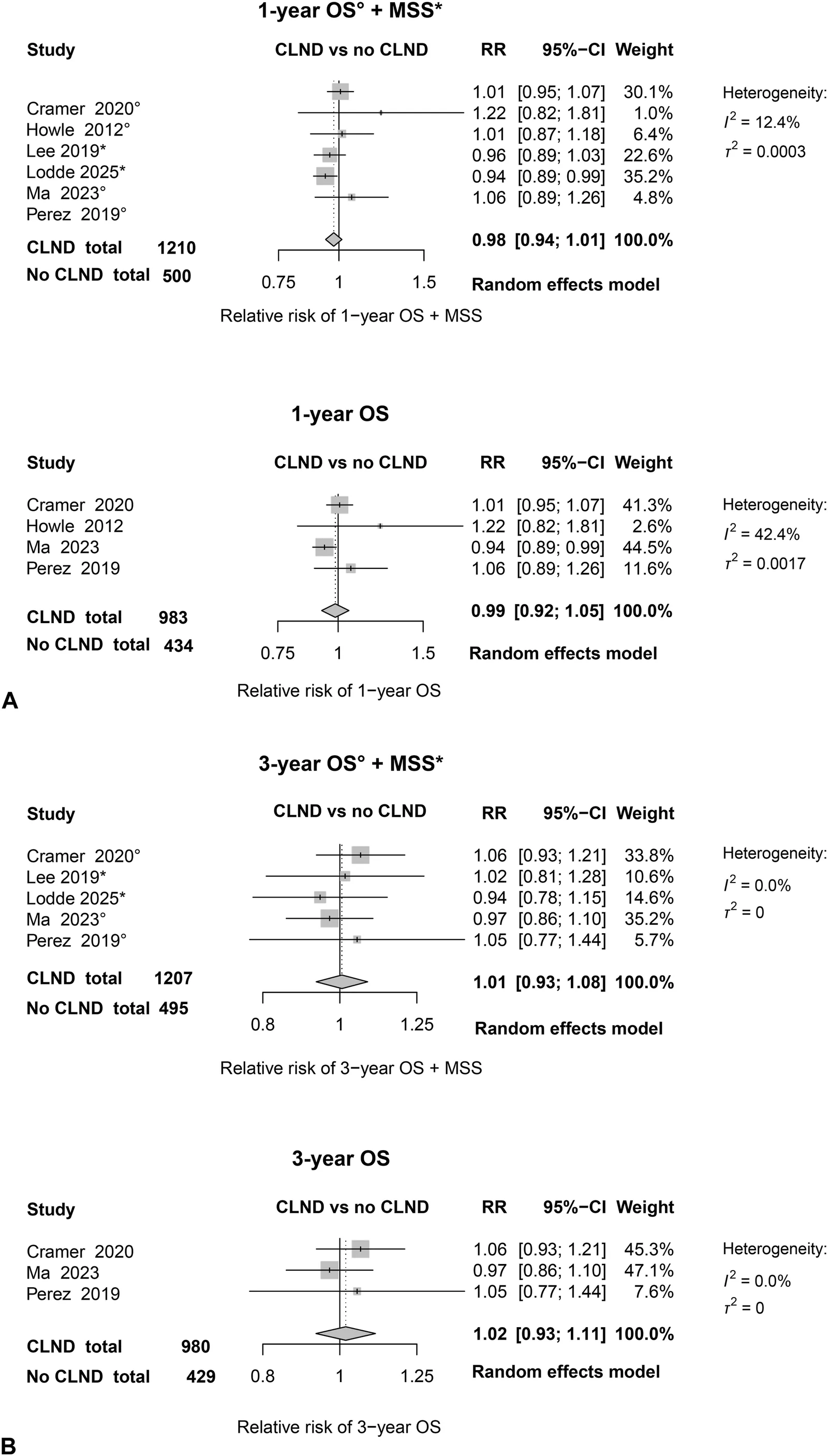
One- and 3-year survival after complete lymph node dissection (CLND) versus no CLND. **(A)** Forrest plots of random-effects (RE) models comparing the RR of 1-year pooled overall survival (OS) and MCC-specific survival (MSS) (upper panel) and 1-year OS (lower panel). **(B)** Forrest plots of RE models for 3-year OS and MSS (upper panel) and 3-year OS (lower panel). Survival rates are presented as RR with 95% CI. Study heterogeneity was evaluated using τ^2^ and I^2^ statistics.

**Table I:** Summary of findings for CLND versus no CLND in patients with stage IIIA Merkel cell carcinoma

Outcomes	No. of participants (studies) Follow-up	Certainty of the evidence	Relative effect (95% CI)	Anticipated absolute effects	
				Risk with no CLND	Risk difference with CLND
1-year NRFS	260 (3 nonrandomized studies)	⨁⨁◯◯ Low∗	RR 1.00 (0.93-1.08)	941 per 1.000	0 fewer per 1.000 (from 66 fewer to 75 more)
3-year NRFS	260 (3 nonrandomized studies)	⨁◯◯◯ Very low†^,^‡	RR 0.98 (0.84-1.16)	906 per 1.000	18 fewer per 1.000 (from 145 fewer to 145 more)
1-year survival	1710 (6 nonrandomized studies)	⨁⨁◯◯ Low†	RR 0.98 (0.94-1.01)	908 per 1.000	18 fewer per 1.000 (from 54 fewer to 9 more)
3-year survival	1702 (5 nonrandomized studies)	⨁⨁◯◯ Low†	RR 1.01 (0.93-1.08)	685 per 1.000	7 more per 1.000 (from 48 fewer to 55 more)

Relative and absolute effects on 1- and 3-year nodal recurrence-free survival (NFRS) and survival are presented as risk ratios (RR) with 95% confidence intervals (95%CI). Certainty of evidence was assessed according to the GRADE framework. Patient or population: patients with stage IIIA MCC; intervention: CLND; comparison: no CLND.

GRADE Working Group grades of evidence: High certainty: we are very confident that the true effect lies close to that of the estimate of the effect. Moderate certainty: we are moderately confident in the effect estimate: the true effect is likely to be close to the estimate of the effect, but there is a possibility that it is substantially different. Low certainty: our confidence in the effect estimate is limited: the true effect may be substantially different from the estimate of the effect.

Very low certainty: we have very little confidence in the effect estimate: the true effect is likely to be substantially different from the estimate of effect.

*CLND*, Complete lymph node dissection; *NRFS*, Nodal recurrence-free survival; *RR*, risk ratio.

∗ The risk in the intervention group (and its 95% CI) is based on the assumed risk in the comparison group and the relative effect of the intervention (and its 95% CI).

† Due to confounding bias and selection bias, we downgraded for one level.

‡ Due to inconsistency between studies, we downgraded for one level.

All analyses showed no evidence of publication bias, and leave-one-out sensitivity analysis confirmed the robustness of the findings. Five-year OS and MSS rates for patients undergoing CLND versus no CLND are approximately 62% and 61%, respectively (Supplementary Fig 2, *A*, available via Mendeley at https://data.mendeley.com/datasets/27ct2dm8x4/1).

### Survival after RTX versus no RTX

To evaluate the effect of RTX on the regional LN station in patients with SLNB-positive MCC, available data were stratified by the performance of RTX. Due to limited data availability, only 1 study reported NRFS without RTX; therefore, the effect of RTX on NRFS could not be assessed. Weighted mean 1-year OS and MSS survival rates were 92% (95% CI, 88%-95%) for patients receiving RTX (with or without CLND) and 87% (95% CI, 84%-89%) for those not receiving RTX and similar values for OS alone (Supplementary Fig 3, *A*, available via Mendeley at https://data.mendeley.com/datasets/27ct2dm8x4/1). Study heterogeneity was low (I^2^ = 41% and I^2^ = 0%). The RE model comparing OS of RTX to no RTX that included 1409 patients showed no significant difference (Fig 3, *A*, Table II). The 3-year OS and MSS survival rate was 69% (95% CI, 62%-77%) for RTX compared with 61% (95% CI, 55%-68%) without RTX, and for OS alone the survival rate was 67% (95% CI, 59%-74%) for RTX compared with 61% (95% CI, 55%-68%) without RTX (Supplementary Fig 3, *B*, available via Mendeley at https://data.mendeley.com/datasets/27ct2dm8x4/1). Risk of bias was moderate (Supplementary Fig 4, *B*, available via Mendeley at https://data.mendeley.com/datasets/27ct2dm8x4/1), and there was no significant difference for OS between RTX and no RTX (χ^2^ = 3.1, *P* = .7). The RE model comparing OS of RTX to no RTX showed no significant difference in RR (Fig 3, *B*, Table II).

**Fig 3:**
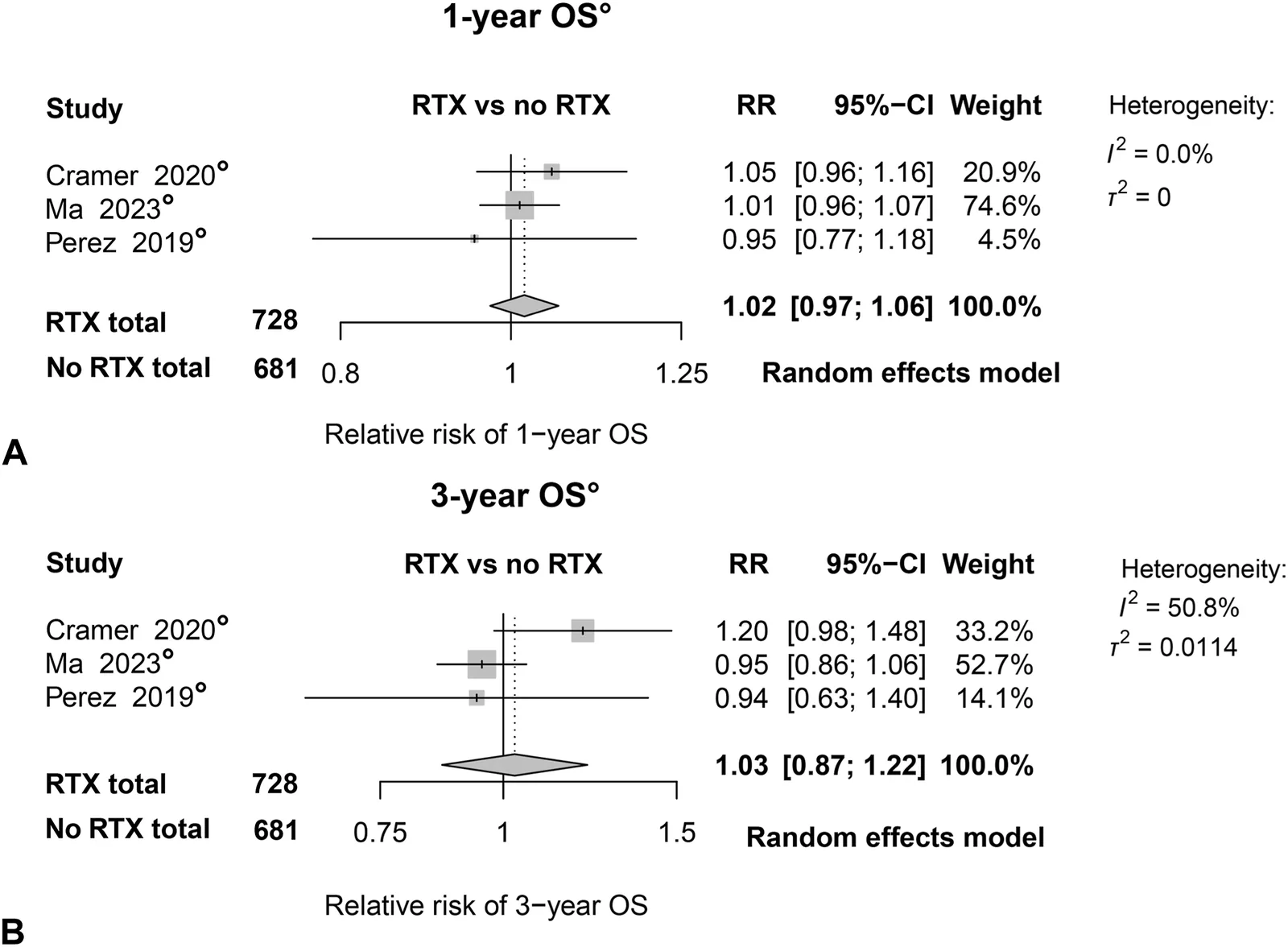
One- and 3-year survival after radiation therapy (RTX) versus no RTX. Forrest plots of RE models comparing the RR of **(A)** 1-year OS and **(B)** 3-year OS. There were no studies reporting MSS. Survival rates are presented as RR with 95% CI. Study heterogeneity was evaluated using τ^2^ and I^2^ statistics.

**Table II:** Summary of findings for radiation therapy (RTX) versus no RTX in patients with stage IIIA MCC

Outcomes	No. of participants (studies) Follow-up	Certainty of the evidence	Relative effect (95% CI)	Anticipated absolute effects	
				Risk with no RTX	Risk difference with RTX
1-year survival	1409 (3 nonrandomized studies)	⨁⨁◯◯ Low∗	RR 1.02 (0.97-1.06)	860 per 1.000	17 more per 1.000 (from 26 fewer to 52 more)
3-year survival	1409 (3 nonrandomized studies)	⨁◯◯◯ Very low∗^,^†^,^‡	RR 1.03 (0.87-1.22)	631 per 1.000	19 more per 1.000 (from 82 fewer to 139 more)

Relative and absolute effects on 1- and 3-year survival are presented as RR with 95% CI. Certainty of evidence was assessed according to the GRADE framework. Patient or population: patients with stage IIIA MCC; intervention: radiation therapy (RTX); comparison: no RTX. The risk in the intervention group (and its 95% CI) is based on the assumed risk in the comparison group and the relative effect of the intervention (and its 95% CI).

GRADE Working Group grades of evidence: High certainty: we are very confident that the true effect lies close to that of the estimate of the effect. Moderate certainty: we are moderately confident in the effect estimate: the true effect is likely to be close to the estimate of the effect, but there is a possibility that it is substantially different. Low certainty: our confidence in the effect estimate is limited: the true effect may be substantially different from the estimate of the effect.

Very low certainty: we have very little confidence in the effect estimate: the true effect is likely to be substantially different from the estimate of effect.

*RR*, Risk ratio.

∗ Due to confounding bias and selection bias, we downgrade for 1 level.

† There was an important inconsistency between studies; 1 study showed a clear survival benefit of RTX (RR >1 with CI entirely above 1), whereas the others showed no clear effect. Confidence intervals of the 2 largest studies did not overlap, and no convincing explanation was identified, so we downgraded 1 level for inconsistency.

‡ Pooled effect estimate (RR, 1.07; 95% CI, 0.83-1.39) is uncertain; the confidence interval includes a clinically important reduction and a clinically important improvement in 1-year survival. Therefore, we downgraded 1 level for imprecision.

For all analyses, there was no indication of publication bias, and leave-one-out sensitivity analysis did not reveal any outlying cohorts. Five-year survival rates for RTX versus no RTX are depicted in Supplementary Figure 2, *B* (available via Mendeley at https://data.mendeley.com/datasets/27ct2dm8x4/1). However, the validity of this analysis is limited due to a high heterogeneity of the studies and the fact that the group without RTX consisted of only 2 studies.

### NMA comparing CLND, RTX, and CLND + RTX

Additionally, a NMA was performed to compare all 3 treatment strategies: CLND, RTX, and CLND + RTX. The interventions were compared using CLND as the reference group. Although the study arms were comparable regarding age and sex, data on other key prognostic factors such as immunosuppression status, number of positive sentinel nodes, and LN basin location were incompletely reported, precluding a detailed transitivity analysis (Supplementary Table II, available via Mendeley at https://data.mendeley.com/datasets/27ct2dm8x4/1). Eight studies reported 1-year survival,^6^^,^^11^^,^^13^^,^^14^^,^^23^^,^^24^^,^^38^^,^^39^ 6 studies reported 3-year survival, and 5 studies reported 5-year survival. The RE model for 1-year OS and MSS survival, respectively for OS survival alone, revealed no significant differences for CLND + RTX or RTX only compared with CLND only (Fig 4, *A*; RR, 1.02 [95% CI, 0.95-1.10]; and RR, 1.03 [95% CI, 0.95-1.11], respectively). Similarly, 3- and 5-year OS and MSS survival did not differ significantly between the interventions (Fig 4, *B*; Supplementary Fig 3, *C*, available via Mendeley at https://data.mendeley.com/datasets/27ct2dm8x4/1). Study heterogeneity was higher for the 3-year survival analysis (I^2^ = 71%) compared with the 1- and 5-year analyses (I^2^ = 39% and I^2^ = 14%, respectively). Overall, study results were broadly consistent across treatments. For 1-year OS and MSS survival, formal tests indicated no substantial disagreement between studies or between direct and indirect comparisons (total Q = 7.47, df = 6, *P* = .28; between-designs Q = 1.05, df = 2, *P* = .59). However, for 3-year OS and MSS survival, heterogeneity increased markedly (I^2^ = 57.4%; Total Q = 11.74, df = 5, *P* = .04), primarily driven by variability within study designs (Q = 11.70, df = 4, *P* = .02), with no meaningful inconsistency between direct and indirect evidence (Q = 0.04, df = 1, *P* = .83). Comparisons of OS rates after excluding the 2 studies that reported only MSS were comparable to the pooled survival estimates (Supplementary Fig 5, available via Mendeley at https://data.mendeley.com/datasets/27ct2dm8x4/1). Sensitivity analyses revealed no substantial differences between the 2 analyses (Supplementary Fig 6, available via Mendeley at https://data.mendeley.com/datasets/27ct2dm8x4/1).

**Fig 4:**
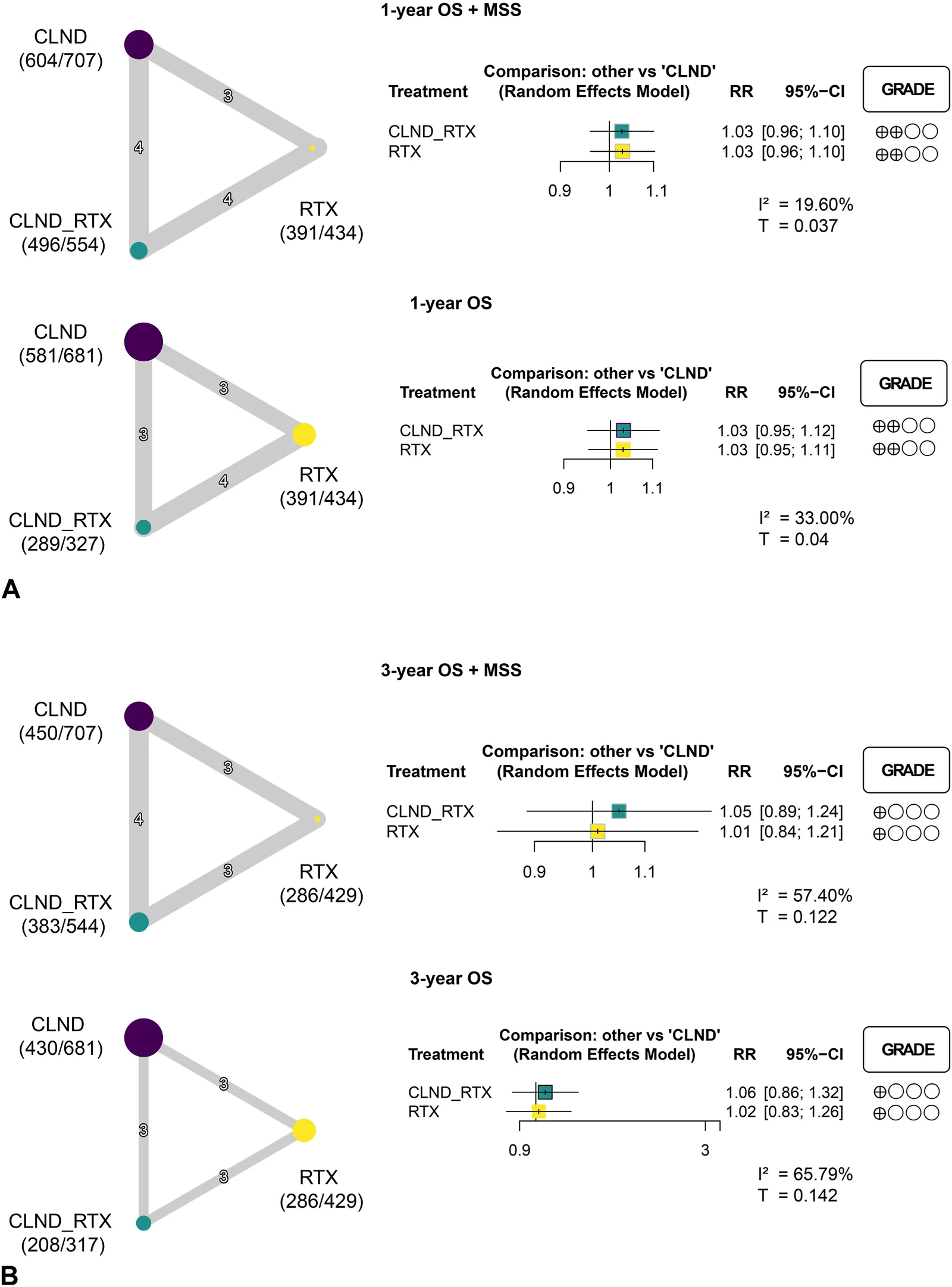
Network meta-analysis comparing RTX alone, and complete lymph node dissection and radiation therapy (CLND + RTX) with CLND alone regarding 1-year **(A)** and 3-year **(B)** pooled OS and MSS survival. On the *left*, the treatment network is displayed as a network graph with node sizes reflecting the total number of patients included in each treatment arm and edge thickness indicating the number of contributing studies. The number of survivors as well as the total number is shown in brackets. On the *right*, forest plots with effect sizes expressed as RR with 95% CI are displayed. τ^2^ and I^2^ are shown as estimates of heterogeneity.

### Data on complications are limited

Beyond survival data, reported complications were extracted. However, data were extremely limited. One study reported 90-day mortality, 30-day readmission rates, and mean length of hospital stay.^13^ Detailed descriptions of specific complications, including lymphedema, surgical-site infections, abscess formation, and postradiation changes, were provided in only one study.^23^

## Discussion

This meta-analysis summarizes survival outcomes following CLND and/or RTX in patients with SLNB-positive MCC. One-year PFS rates across included studies ranged from 67.6% to 100%, while 1-year NRFS ranged from 84.1% to 100%. The divergence between PFS and NRFS rates indicates that recurrence at distant sites may contribute to progression and is not fully addressed by local treatments such as CLND and RTX. One-year survival rates were high, at 93% (95% CI, 89%-96%) for patients receiving CLND (with or without RTX) and 92% (95% CI, 88%-95%) for patients receiving RTX (with or without CLND) and RR comparison, as well as NMA comparing CLND, RTX, and CLND + RTX showed no significant survival differences (certainty of evidence: low to very low).

However, moderate between-study heterogeneity might be explained by potential unmeasured confounding factors, such as performance status or comorbidities leading to treatment selection. Furthermore, the timing of adjuvant RTX is pivotal, as survival benefits were observed only when RTX was initiated within 8 weeks of diagnosis.^6^ However, most studies do not specify the timing of RTX initiation, precluding any comparison of outcomes based on treatment timing.

Since CLND + RTX did not confer an additional survival advantage over CLND alone or RTX alone (certainty of evidence: low to very low), clinicians may consider individualized treatment decisions recognizing that performing CLND may postpone the initiation of RTX and thereby be associated with a less favorable prognosis.^6^ Although data specific to patients with stage IIIA MCC are limited, RTX is generally associated with a lower risk of lymphatic drainage disorders and avoids operation-related morbidity.^21^^,^^22^ Given that elderly patients may particularly benefit from RTX. However, in this meta-analysis, morbidity outcomes could not be evaluated because only two studies reported on any morbidity parameters; consequently, evidence-based comparisons of morbidity within this analysis are not feasible.

Although 1-year survival rates were high regardless of the adjuvant treatment, 5-year survival rates ranged only ∼50%. The differing NRFS and PFS rates support the consideration of MCC as a systemic disease in many patients. ICI have proven efficiency in advanced MCC,^40^, ^41^, ^42^, ^43^ but their benefit in the adjuvant setting remains unproven.

Despite including ICI terms in our search strategy, insufficient data were available on adjuvant ICI in stage IIIA MCC for quantitative synthesis and inclusion in the analysis. Therefore, the present meta-analysis does not provide any evidence to guide the sequencing of therapies or the integration of immunotherapy with local modalities, a question of considerable clinical relevance. While the ADMEC-O phase II trial of adjuvant nivolumab versus observation in completely resected patients with MCC (stages I–IV) showed improved disease-free survival, the difference was not statistically significant.^44^ Specifically, in the stage IIIA subgroup, disease-free survival did not differ between patients receiving adjuvant nivolumab and observation (25 and 8 patients, respectively).^44^ Unfortunately, the ADMEC-O trial did not report whether stage IIIA patients received additional treatments such as CLND alone, RTX alone, or CLND + RTX, precluding inclusion of the study in the NMA. Besides, the study’s limited statistical power, owing to its small sample size, restricts the strengths of its conclusions. Nonetheless, patients with stage IIIA MCC may still benefit from adjuvant immunotherapy. Consequently, larger clinical trials enrolling stage IIIA patients are required. If adjuvant therapy confers a survival advantage, the systemic efficacy of immunotherapy could obviate the need for local therapies such as CLND or RTX. Currently, evidence regarding the benefit of RTX, CLND, or their combination in the context of adjuvant immunotherapy is lacking. For clinically detected regional LN metastasis, Chang et al have already demonstrated significantly improved OS with neoadjuvant immunotherapy.^45^ However, the complete pathologic nodal response rate was low, at only 17.9%,^45^ suggesting that local treatments are still necessary. The only trial investigating combined immunotherapy and RTX included only patients with advanced MCC showing no additional benefit from stereotactic body radiation therapy compared to dual immunotherapy alone.^46^ For stage IIIA patients, however, optimal therapy combinations including immunotherapies and local therapies such as CLND, RTX, or CLND + RTX remain undefined and desperately require further research. The ADAM trial (NCT03271372) is currently studying the benefit of avelumab in patients with MCC and LN metastasis who have undergone surgery and/or radiation therapy. Subgroup analysis of stage IIIA patients will hopefully help improve the evidence regarding this pressing research question.

## Limitations

The conclusions from this meta-analysis are limited by several factors. First, the limited availability of data, due to few studies and partially small sample sizes, potentially reduced the statistical power of the analyses. The limited data availability is likely attributable to the rarity of MCC (<0.3 cases/100,000/y^3^). Consequently, subgroup analyses, eg, in high-risk groups such as patients with immunosuppression, male sex, or older age^15^ were not feasible, and the limited number of studies precluded formal meta-regression analyses. Furthermore, heterogeneous outcome reporting across studies also restricted data availability. Therefore, the effects of the interventions on PFS and NRFS could not be investigated. Besides, 2 studies reported only MSS and others only OS. Because MSS rates approximated OS rates, a combined survival parameter was calculated to increase sample size in the RE model. However, this combined parameter introduces a competing-risk bias, which may reduce interpretability despite showing no substantial differences compared with OS-only models. MSS is a disease-specific end point that excluded deaths from causes unrelated to MCC, whereas OS records death from any cause—a particularly relevant distinction in the relatively elderly MCC population. In retrospective studies, MSS may be biased, potentially leading to an overestimation of the effect of the interventions on survival. Pooling these 2 different survival outcomes introduces heterogeneity, may distort effect estimates, and may inflate the apparent benefit of the treatments. Six of the included studies reported OS while only 2 provided MSS. Therefore, OS data dominate this meta-analysis.

Heterogeneous reporting encompassed not only outcome parameters but also patient characteristics, occurrence of complications, and treatment protocols such as the interval between CLND and RTX initiation. Transitivity analysis was only assessed based on sex and age, while important effect modifiers, including immunosuppression status and number of positive lymph nodes, could not be considered due to limited data availability. This challenges the transitivity assumption and markedly limits the interpretation of the NMA results.

Moreover, the retrospective nature of the included studies limited the evidence level to a maximum of “low” according to the GRADE criteria. In some cases, the certainty of evidence was graded as “very low” because of a high risk of confounding or imprecision. As noted above, confounding is likely a substantial source of bias in this analysis, restricting the findings to associative observations and precluding causal conclusions. Combined with the limited reporting of treatment-related morbidities and ICI regimes, this low evidence level strongly supports the need for standardized prospective trials to evaluate the prognostic role of subsequent therapies in SLNB-positive MCC.

## Conclusion

The NMA demonstrated comparable survival outcomes with CLND only, RTX only, and CLND + RTX in patients with SLNB-positive MCC (certainty of evidence: low to very low). Given the low certainty of the available evidence at the moment, we recommend that treatment decisions be individualized based on each patient’s comorbidities and preferences, with CLND alone, RTX alone, and the combination CLND + RTX considered on a case-by-case basis. This meta-analysis underscores the urgent need for high-quality prospective trials, including studies with additional adjuvant immunotherapies, to enable definitive recommendations.

## Conflicts of interest

Dr Wasserer received speaker’s honoraria/travel support from Janssen, Sanofi, Almirall, Novartis, and Lilly. Dr Preis received speaker’s honoraria from Janssen, Novartis, and AbbVie. Dr Biedermann received grants from Almirall, Celgene-BMS, Lilly, Novartis, Sanofi-Genzyme, Regeneron, and Viatris; received consulting fees from AbbVie, Alk-Abello, Almirall, Boehringer-Ingelheim, Leo Pharma, Lilly, Novartis, Sanofi-Genzyme, and Viatris; received honoraria from Alk-Abello, Almirall, GSK, Leo Pharma, Lilly, Novartis, Sanofi-Genzyme, and Regeneron; and is a member of the advisory board of Alk-Abello, Almirall, Boehringer-Ingelheim, Leo Pharma, Lilly, Novartis, Sanofi-Genzyme, and Viatris. Dr Persa received personal honoraria from Merck Sharp & Dohme; received travel support from Kyowa Kirin, Pierre Fabre, Sanofi, and Sun Pharma; and is a member of the advisory board of Bristol Myers Squibb, Almirall, and Sanofi. Dr Mayer received travel support from Novartis. Drs Foerster and Lauffer have no conflicts of interest to declare.
